# Structural Identification of Ginsenoside Based on UPLC-QTOF-MS of Black Ginseng (*Panax Ginseng* C.A. Mayer)

**DOI:** 10.3390/metabo14010062

**Published:** 2024-01-18

**Authors:** Hyo-Bin Oh, Da-Eun Jeong, Da-Eun Lee, Jong-Hee Yoo, Young-Soo Kim, Tae-Young Kim

**Affiliations:** 1 Institute of Jinan Red Ginseng, Jinan-gun 55442, Republic of Korea; dmsghktn333@ijrg.re.kr (D.-E.J.); lee819@ijrg.re.kr (D.-E.L.); jonghee79@ijrg.re.kr (J.-H.Y.); kty54090@ijrg.re.kr (T.-Y.K.); 2 Department of Food Science and Technology, Jeonbuk National University, Jeonju 54896, Republic of Korea; ykim@jbnu.ac.kr

**Keywords:** white ginseng, red ginseng, black ginseng, steaming process, metabolomics, Maillard reaction, ginsenoside

## Abstract

Black ginseng (BG) is processed ginseng traditionally made in Korea via the steaming and drying of ginseng root through three or more cycles, leading to changes in its appearance due to the Maillard reaction on its surface, resulting in a dark coloration. In this study, we explored markers for differentiating processed ginseng by analyzing the chemical characteristics of BG. We elucidated a new method for the structural identification of ginsenoside metabolites and described the features of processed ginseng using UPLC-QTOF-MS in the positive ion mode. We confirmed that maltose, glucose, and fructose, along with L-arginine, L-histidine, and L-lysine, were the key compounds responsible for the changes in the external quality of BG. These compounds can serve as important metabolic markers for distinguishing BG from conventionally processed ginseng. The major characteristics of white ginseng, red ginseng, and BG can be distinguished based on their high-polarity and low-polarity ginsenosides, and a precise method for the structural elucidation of ginsenosides in the positive ion mode is presented.

## 1. Introduction

In South Korea, processed ginseng is represented by three main types: white ginseng (WG), red ginseng (RG), and black ginseng (BG) [1,2]. WG is manufactured by drying ginseng without undergoing a steaming process, and it is characterized by its bright color. In contrast, RG is steamed once and then dried, resulting in a red color.

BG is traditionally made in South Korea by repeatedly steaming and drying ginseng at least three times. During the repeated steaming process, the outer surface darkens due to the Maillard reaction, giving it its characteristic dark color. Ginseng primarily contains over 30% starch and approximately 12% protein [3]. When ginseng is processed using steam, it undergoes the Maillard reaction, leading to browning, which is attributed to reducing sugars from starch and amino-carbonyl reactions from proteins [4,5].

The major component in BG, ginsenosides, is associated with various physiological activities. Through the repeated steaming process, polar ginsenosides decrease. Conversely, less polar ginsenosides gradually increase as a result of the repetitive steaming process. The main reactions, including hydrolysis, dehydration, and isomerization reactions, occur at C-20 of the ginsenosides. Hydrolysis primarily takes place at C-3 and C-6 [6].

BG contains minor ginsenosides such as G-Rg3, G-Rk1, and Rg5, which are not present in unprocessed ginseng [7,8]. These components are associated with various physiological functions, including anticancer, fat reduction, and anti-inflammatory effects [9,10,11].

Research on the active ingredients of BG is diverse and ongoing. However, there is limited research on the transformation process from ginseng to BG. Additionally, while there have been numerous studies on ginseng and RG metabolism, research on BG is lacking [12,13].

In this study, we aimed to explore the components that can determine the quality of BG and elucidate their correlations. We describe the representative markers of BG through a metabolomic approach to the major active ingredients.

## 2. Materials and Methods

### 2.1. Materials and Equipment

Ginseng used as the raw material to manufacture the BG samples was 5-year-old ginseng harvested in November 2022. The harvested ginseng was used to process BG after removing foreign substances using a ginseng washing machine. The dried BG was ground using a grinder and sieved to a 100-mesh size for use as the sample.

The solvents used in this study included methanol (high-performance liquid chromatography (HPLC)-grade, Duksan, Ansan-si, Republic of Korea), tertiary distilled water, acetonitrile (ACN, HPLC-grade, J.T Baker, Phila, PA, USA), ethanol (HPLC-grade, Duksan), hexane (HPLC-grade, Duksan), and dichloromethane (HPLC-grade, Duksan). Benzo[a]pyrene standards and internal standards were prepared by dissolving benzo[a]pyrene (98%, Sigma-Aldrich Co., Darmstadt, Germany) and 3-methylcholine (100 ppm, Sigma-Aldrich Co.) in ACN at a concentration of 1 µg/mL, followed by serial dilution. Free amino acid 17S standard mixtures (2.5 μmol/mL, Merck Co., Darmstadt, Germany) were used after serial dilution in 0.1 N HCl (Merck Co., Germany). The free sugar standards included glucose (99.9%, Sigma-Aldrich Co.), fructose (99%, Sigma-Aldrich Co.), maltose (99%, Sigma-Aldrich Co.), xylose (99%, Sigma-Aldrich Co.), rhamnose (99%, Sigma-Aldrich Co.), arabinose (99%, Sigma-Aldrich Co.), and sucrose (99.9%, Sigma-Aldrich Co.). They were dissolved in distilled water to a concentration of 1 mg/mL, followed by serial dilution.

Analysis was performed by HPLC-refractive index detection (RID; Agilent, Santa Clara, CA, USA), fluorometric detection (FLD; Agilent) using an Agilent 1200 series HPLC system, liquid chromatography with tandem mass spectroscopy (LC-MS/MS) system (QTRAP 4500, SCIEX, Pte, Ltd., Toronto, ON, Canada), a color difference meter (KONICA MINOLTA SENSING, Inc., Tokyo, Japan), and ultrasonic extraction device (JEIO TECH, UC-20, Co., Daejeon, Republic of Korea). For metabolite analysis, ultra-high-performance liquid chromatography-quadrupole time-of-flight mass spectrometry (UPLC-QTOF-MS; SCIEX, Pte, Ltd.), nitrogen concentrator (CHONGMIN TECH, Seoul, Republic of Korea), and solid phase extraction (LABTECH, Namyangju-si Republic of Korea) were used.

### 2.2. Steam Processing and Appearance Observation

**Figure 1:**
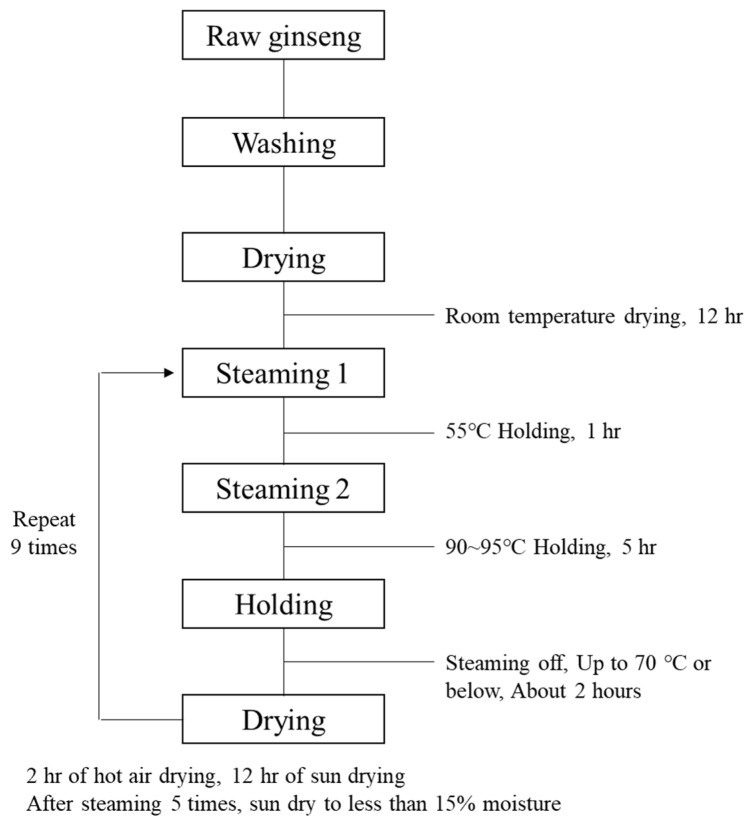
Black ginseng production process.

Ginseng was dried in a drying facility at 40 °C for 6 h to prevent cracking during steaming, left at room temperature for 12 h, and then used to produce BG. The steaming process is shown in Figure 1.

Lightness (L), redness (a), and yellowness (b) were quantified on the surface of the ginseng sample using a colorimeter. Each manufactured sample was measured three times.

### 2.3. Sample Preparation

#### 2.3.1. Benzo[a]pyrene

BAP and 3-methylcholinerene standards were each dissolved in ACN to a concentration of 1 μg/mL. Appropriate amounts of standard and internal standards were accurately taken and diluted with ACN to prepare 3, 5, 10, 20, and 40 ng/mL of BAP and 50 ng/mL of internal standard. Then, 5.0 g of each sample was precisely weighed. After adding 100 mL of water, ultrasonic extraction was performed for 90 min. After adding 100 mL of hexane and 1 mL of internal standard solution, the mixture was homogenized for 5 min, followed by ultrasonic extraction for 30 min. The hexane layer was transferred to a separatory funnel. After adding 50 mL of hexane to the water layer, the mixture was shaken twice, and the hexane layer was taken and combined in the separatory funnel. After adding 50 mL of water to the hexane layer and washing, the hexane layer was dehydrated, filtered using a filter paper containing sodium sulfate, and then concentrated under reduced pressure in a water bath at 45 °C until about 2 mL of hexane remained. A Florisil cartridge was used after 10 mL of dichloromethane and 20 mL of hexane were sequentially activated by addition at a rate of 2–3 drops per second. The extract was placed in an activation cartridge. Then, 20 mL of a mixture of hexane and dichloromethane (3:1) was eluted at a rate of 2–3 drops per second. The eluted solution was concentrated under nitrogen gas in a water bath at 35 °C. The residue was dissolved in 1 mL of ACN and filtered through a membrane filter of 0.20 μm or less to prepare the test solution [14].

#### 2.3.2. Free Sugar

Each saccharide standard product (fructose, glucose, sucrose, maltose, arabinose, and rhamnose) was dried for 12 h, dissolved in distilled water, mixed, and used to prepare serial dilutions. For each test solution, 0.5 g of the homogenized sample was precisely weighed. Then, 25 mL of 50% MeOH was added, refluxed at 85 °C, and cooled to room temperature. In the case of turbidity, the supernatant was centrifuged for 10 min and then filtered using a 0.45 μm filter to prepare the test solution [15].

#### 2.3.3. Free Amino Acid

Herbert’s method was used for free amino acid preparation [16]. Briefly, 0.1 g of the sample was taken into a Teflon capping test tube, 10 mL of 6 N hydrochloric acid was added, nitrogen gas was blown, and the sample was hydrolyzed for 3 h in an autoclave at 121 °C. The hydrolysate was filtered through a 0.45 μm PTFE filter, and impurities were removed using a C18 cartridge and then used as the sample solution.

#### 2.3.4. Ginsenoside Metabolite

The sample (1 g) was placed in a 50 mL conical tube with 9 mL of 70% MeOH and shaken. Ultrasonic extraction was performed for 30 min [17]. The extract was centrifuged (4000 rpm, 10 min) and filtered through a 0.2 μm syringe filter. Afterward, an SPE cartridge (C18, 2000 mg) was used, which was activated in order with 5 mL of methanol and 5 mL of water, loaded with 1 mL of the filtrate, and then washed with 5 mL of water. After additional washing with 10 mL of methanol, the eluate was concentrated with nitrogen. The concentrate was re-dissolved in 0.5 mL of each extraction solvent, filtered through a 0.2 μm syringe filter, and then injected into the device for analysis.

### 2.4. Analysis of BAP by HPLC-FLD

An Agilent 1200 series HPLC system equipped with an FLD detector and an Eclipse Plus C18 column (4.6 × 150 mm, 3.5 µm) was used to analyze the BAP content. The injection amount was 10 μL. The flow rate was set at 1.0 mL/min. The column temperature was maintained at 30 °C. The excitation and fluorescence wavelengths were set at 294 and 404 nm, respectively, for the analysis. A gradient elution of solvent A (water) and solvent B (ACN) was used as follows: 0–30 min, 30% A and 70% B; 30–31 min, 5% A and 95% B; 31–36 min, 5% A and 95% B; 36–37 min, 30% A and 70% B; 37–45 min, 30% A and 70% B.

### 2.5. Analysis of Free Sugar by HPLC-RID

An Agilent 1200 series HPLC system equipped with an RID detector was used to analyze free sugar content. ZORBAX carbohydrate (4.6 × 250 mm, 5 µm) was used in the column. The injection amount was 10 μL. The flow rate was 1.0 mL/min, and the column temperature was 35 °C. The mobile phase was analyzed for 30 min using water (A) and ACN (B) at a ratio of 75:25 (*v*:*v*).

### 2.6. Analysis of free Amino Acids by UPLC-MS/MS

A QTRAP 4500 series MS/MS system (SCIEX, USA) equipped with a UPLC (Agilent) system and Intrada IMTAKT amino acid column (3.0 × 150 mm, 3.0 µm) was used to analyze the amino acid content. The injection amount was 2 μL. The flow rate was set at 0.3 mL/min. The column temperature was maintained at 40 °C. A gradient elution of solvent A (water) and solvent B (ACN) was used as follows: 0–4 min, 20% A and 80% B; 4–14 min, 0% A and 100% B; 14–16 min, 0% A and 100% B; 16.1–20 min, 80% A and 20% B.

The MS analysis conditions used the positive ion mode. The curtain gas and collision gas were set to 30 and 3, respectively. The ion spray voltage and ion source temperature were set to 5500 and 550, respectively.

### 2.7. Analysis of Ginsenoside Metabolite by UPLC-QTOF-MS

An X500R series QTOF-MS system (SCIEX, Pte, Ltd.) equipped with a UPLC (Agilent) system and CORETECS T3 column (2.1 × 150 mm, 1.6 µm, Waters, Milford, MA, USA) was used to analyze ginsenoside metabolites. The injection amount was 1 μL. The flow rate was set at 0.35 mL/min. The column temperature was maintained at 30 °C. A gradient elution of solvent A (0.1% formic acid + water) and solvent B (0.1% formic acid + ACN) was used as follows: 0–10 min, 85% A and 15% B; 10–40 min, 50% A and 50% B; 40–45 min, 50% A and 50% B; 45–50 min, 30% A and 70% B; 50–60 min, 5% A and 95% B; 60–65 min, 85% A and 15% B; 65–80 min, 85% A and 15% B.

QTOF-MS analysis was conducted in the TOF-MS mode with a mass range of 100–1500 *m*/*z*. The ion source gas and curtain gas were set to 50 and 30, respectively. The ion source temperature was maintained at 500 °C. The declustering potential (DP) and collision energy (CE) values were set to 50 and 15 V, respectively. The spray voltage and CE spread were adjusted to 4500 and 10 V, respectively. The analysis was performed in the positive mode.

### 2.8. Statistical Analysis

Statistical analysis was conducted using the SPSS 18 (SPSS Inc., Chicago, IL, USA) program, and a one-way analysis of variance was performed to assess the significance of differences among means (*p* < 0.01). Multiple range testing using Duncan’s method was employed to compare significant differences. Multivariate analysis, including principal component analysis (PCA) and partial least squares-discriminant analysis (PLS-DA), was conducted using MarkerView 1.3.1 (AB Sciex, Pte, Ltd., Toronto, ON, Canada) and MetaboAnalyst 5.0 (https://www.metaboanalyst.ca/, accessed on 18 August 2020). For visualization, the data were analyzed using a heatmap, and significance was determined at the *p* < 0.05 level.

## 3. Results

### 3.1. Appearance Change Characteristics of BG

**Figure 2:**
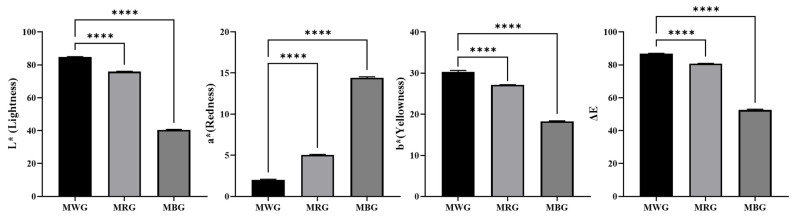
Comparison of color differences between white ginseng, red ginseng, and black ginseng; MWG: Main root white ginseng; MRG: Main root red ginseng; MBG: Main root black ginseng; The data are expressed as the mean ± SD (n = 3); **** *p* < 0.0001, compared to MWG.

WG and RG were used as control groups to compare the external characteristics of BG. The color difference values are as shown in Figure 2.

The color difference measurement results indicated a decrease in brightness, represented by the ΔL value, and an increase in redness, represented by the Δa value. Yellowness, indicated by the Δb value, decreased. Ginseng’s reducing sugars were reported to be decomposed into 5-hydroxymethyl-2-furaldehyde (5-HMF) during BG production. Following this, 5-HMF then combines with amino acids and proteins to generate melanoidins, the final products of the Maillard reaction, thus promoting browning [18].

The results of free sugars that affect the change in appearance quality by BG processing are shown in Table 1.

WG exhibits high levels of sucrose and maltose, indicating a state where no physicochemical reactions have occurred due to the absence of the steaming process. When processed into RG, sucrose decreased by approximately 50%, while fructose and glucose levels remained unchanged. Since sucrose typically undergoes hydrolysis to fructose and glucose, the observed lack of increase in sucrose levels in RG suggests its utilization in browning reactions. In a study by Kim et al., it was reported that when sucrose was steamed at 100 °C for more than 30 h, 95% of sucrose was hydrolyzed into glucose and fructose and used for browning reaction [19]. Maltose was found to have the highest content in WG and decreased by over 80% when processed into BG. Maltose is considered to be a disaccharide consisting of two glucose units that can be hydrolyzed into glucose and subsequently utilized in browning reactions. Jee et al. has reported an increasing trend in the presence of rhamnose and xylose in non-cellulosic carbohydrates under the influence of organic acids and high-temperature conditions [20]. In our study, it is also presumed that these sugars increase due to repeated maturation processes and the rise in organic acids. Additionally, ginsenosides in ginseng contain various sugars, such as glucose, xylose, arabinose, and rhamnose, attached to the non-reducing ends of saponin structures [21]. These sugars are known to be unstable under conditions of heat, acid, or alkali, resulting in their decomposition. However, rhamnose and xylose are considered to have no impact on the browning reactions in BG.

The free amino acid results are shown in Table 2.

When compared to the control groups, WG and RG, the major reduced amino acid contents were identified in the following order: L-arginine, L-lysine, and L-histidine. In previous studies by Do and others, the sugars that promoted ginseng browning reactions were maltose, glucose, and fructose, following the same order [22]. This study yielded ginseng amino acid results consistent with those studies, showing that arginine, histidine, and lysine promoted browning reactions [22].

Free sugars can undergo dehydration and rearrangement reactions to generate 5-HMF from hexoses [23]. Significantly, 5-HMF serves as an intermediate compound in the Maillard reaction and acts as a precursor to melanoidins, which are the final products of this reaction [18]. This conversion is notably promoted under acidic conditions and is primarily observed in traditional herbal medicines like black ginseng. It is acknowledged as an antioxidant compound that tends to increase with prolonged high temperature and repetitive heat treatments [24].

Therefore, reducing sugars and reducing amino acids can be utilized as metabolic markers to distinguish between BG, WG, and RG, and browning compounds, such as melanoidins and 5-HMF, can also be used as markers.

### 3.2. Analysis of BG Ginsenoside Metabolites

In the metabolic analysis of ginseng species, mass spectral patterns, primarily negative ions, have been emphasized as key for structural elucidation. Negative ion patterns offer the advantage of high instrument sensitivity, allowing for detection even at low concentrations. In positive ion patterns, hydrogen ions (H^+^, *m*/*z* 1.007), ammonium ions (NH_4_^+^, 18.03 *m*/*z*), sodium ions (Na^+^, *m*/*z* 22.99), and potassium ions (K^+^, *m*/*z* 38.96) can be additionally detected. This facilitates the straightforward differentiation of ginsenoside intact molecules and the accurate confirmation of fragment ion patterns as sugars are removed. In this study, a total of 79 ginsenosides were structurally elucidated, and their quantitative composition was predicted based on ion intensity (Table 3).

**Figure 3:**
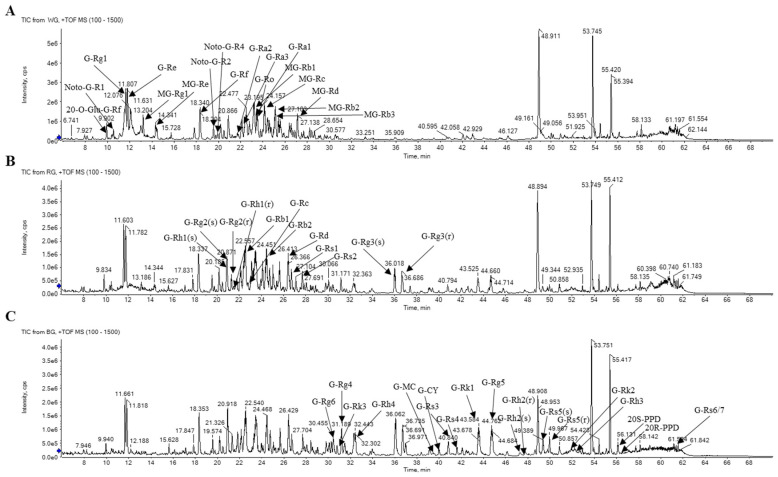
Ginsenoside total ion chromatogram of WG, RG, and BG using UPLC-QTOF-MS.; (**A**): White ginseng; (**B**): Red ginseng; (**C**): Black ginseng.

The total ion chromatograms (TIC) of major ginsenosides found in WG, RG, and BG are shown in Figure 3.

The TIC of major ginsenosides observed in WG, RG, and BG are presented in the following figure. In the case of WG, the more polar ginsenoside components are prominent in the 0–20 min region, characterized by a higher proportion of solvent A (water). For RG, polar ginsenosides decreased, while less polar ginsenosides became more prominent in the 30–50 min range where solvent B (ACN) predominated. BG exhibited an increased presence of less-polar ginsenoside components compared to RG.

**Figure 4:**
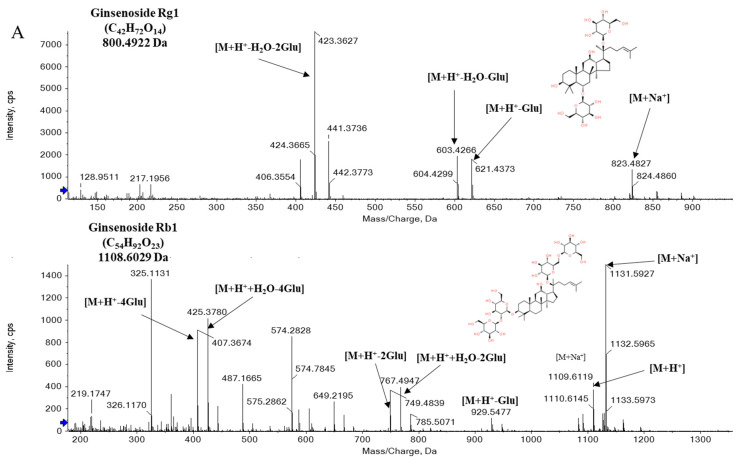

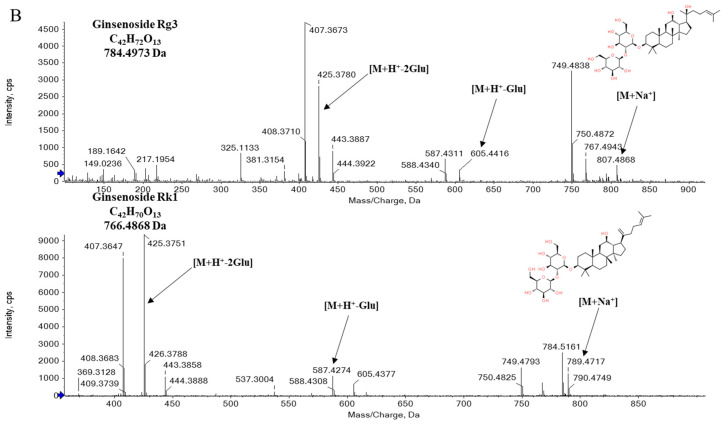
Identification of ginsenosides by UPLC-QTOF-MS in the positive ion mode; (**A**): Ginsenoside Rg1, Ginsenoside Rb1; (**B**): Ginsenoside Rg3, Ginsenoside Rk1; Na^+^: 22.9898 Da, H^+^: 1.0078 Da, Glu: 180.0634 Da (C_6_H_12_O_6_)_._

The structural identification of ginsenosides in BG is shown in Figure 4.

In the structural identification of ginsenoside, sodium ions and ammonium ions showed a pattern that could identify the parent molecule. For example, the exact mass parent molecule value of G-Rg1 (C_42_H_72_O_14_) was at *m*/*z* 800.4922. When a sodium ion is attached to the parent molecule, the theoretical value is 823.4819, and the mass value observed in this study was *m*/*z* 823.4827, which was an accurate structural identification with a ppm error within 0.1. G-Rg1 has a structure with a total of two glucose, each attached at C-6 and C-20. In conclusion, the pattern in which water molecules (H_2_O) were removed and the pattern in which glucose was removed from the parent structure was confirmed.

**Figure 5:**
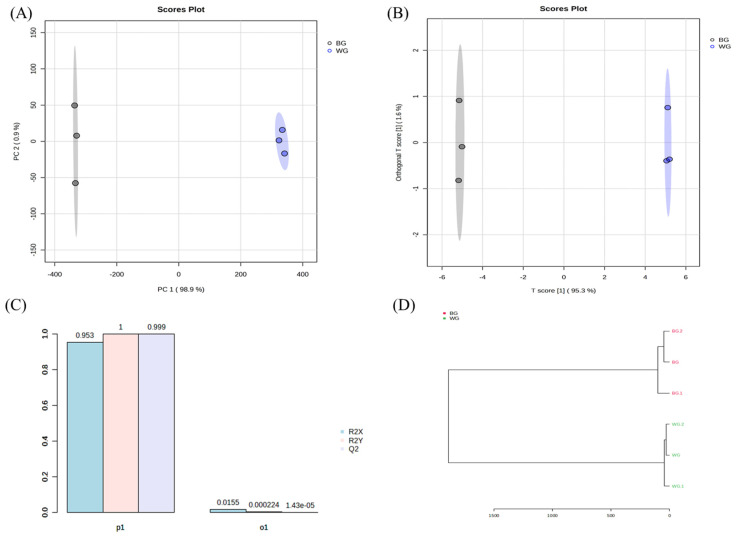
Analysis of main root black ginseng and white ginseng saponin metabolomics data obtained from UPLC-QTOF-MS. (**A**) PCA and (**B**) PLS-DA score plots. (**C**) Modeling parameters. (**D**) HCA dendrogram.

The comparison of WG and BG used PCA (Principal Component Analysis) and OPLS-DA (Orthogonal Partial Least Squares Discriminant Analysis) analysis to compare metabolites. The results are shown in Figure 5.

PCA is typically employed when all variable attributes are denoted as X, while PLS and OPLS are used when the variable attributes are separated into X and Y. X is generally designated as the variable controlling the process, and Y is utilized to specify the numerical values representing the outcomes influenced by X [25].

The PCA values for PC1 and PC2 were 98.9% and 0.9%, respectively, signifying the separation of metabolites between WG and BG. Subsequently, an OPLS-DA was conducted based on the PCA results. Orthogonal T scores 1 and 2 represented 1.6% and 95.3%, respectively, amounting to a total of 96.9%. This clearly indicates the segregation of ginsenoside metabolites between WG and BG.

In the context of the VIP score, a value of 1.0 or higher is commonly considered to indicate an important variable, while a score of 0.8 or lower is not deemed significant [26].

**Figure 6:**
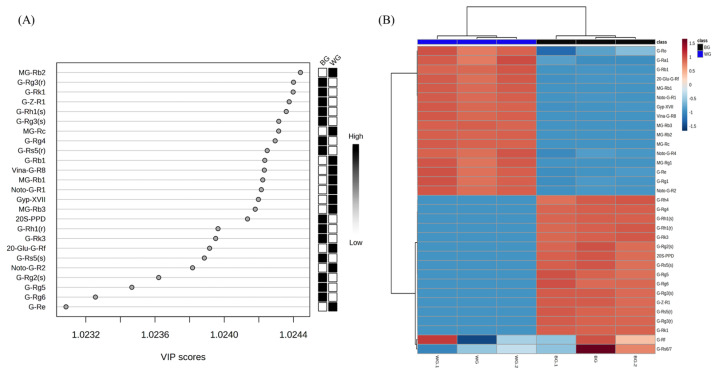
Analysis of main root black ginseng and white ginseng saponin metabolomics data obtained from UPLC-QTOF-MS. (**A**) VIP scores. (**B**) Heat map and HCA clustering results.

The results of extracting ginsenoside metabolites with a VIP (Variable Importance in Projection) score of 1.0 or higher from the OPLS-DA models for WG and BG, along with variables showing a *p*-value of less than 0.01 in the T-test, are shown in Figure 6.

According to the VIP score analysis, the major ginsenosides present in WG included malonyl ginsenoside (MG) Rg1, MG-Rb1, MG-Rc, MG-Rd, ginsenoside(G) Rg1, G-Ra1, G-Re, G-Ro, G-Rb1, notoginsenoside (NG) R1, NG-R2, and 20-O-glucoginsenoside Rf. These compounds serve as discernible markers for differentiation. These results were visualized using heatmap analysis.

**Figure 7:**
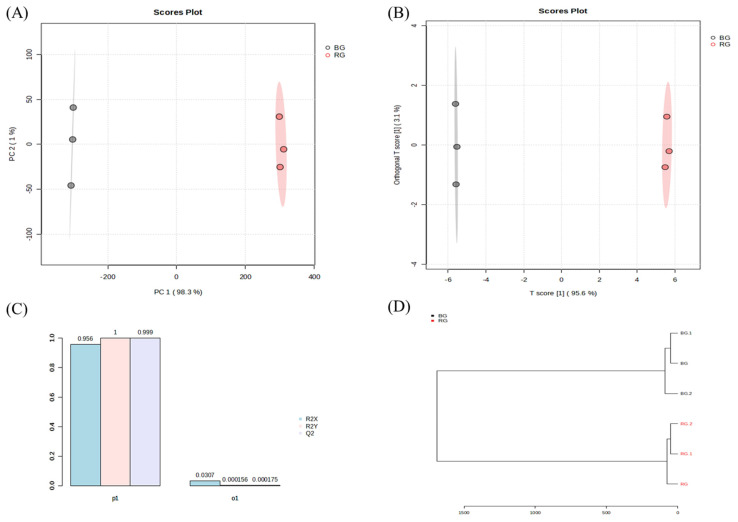
Analysis of main root black ginseng and red ginseng saponin metabolomics data obtained from UPLC-QTOF-MS. (**A**) PCA and (**B**) PLS-DA score plots. (**C**) Modeling parameters. (**D**) HCA dendrogram.

RG and BG were compared using the same method, and the results are shown in Figure 7.

The PCA analysis yielded PC1 and PC2 values of 98.3% and 1%, respectively. This result indicates the separation of metabolites between RG and BG. Subsequently, an OPLS-DA analysis was performed based on the PCA results. Orthogonal T scores 1 and 2 showed values of 3.1% and 95.6%, respectively. In total, these values accounted for 98.7%, confirming the differentiation of ginsenoside metabolites between RG and BG.

**Figure 8:**
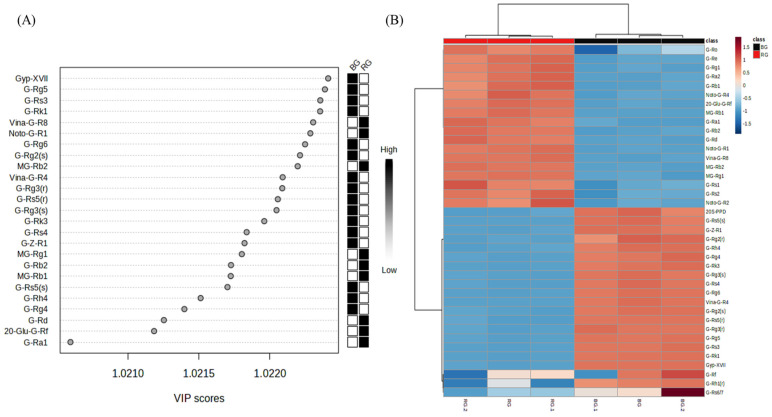
Analysis of main root black ginseng and red ginseng saponin metabolomics data obtained from UPLC-QTOF-MS. (**A**) VIP scores. (**B**) Heat map and HCA clustering results.

The results of extracting ginsenoside metabolites from the OPLS-DA model of RG and BG for significant variables with a VIP score of 1.0 or more and *p*-values from the T-test of less than 0.01 are shown in Figure 8.

A total of 98.7% separation was confirmed between RG and BG in terms of ginsenoside metabolites. According to the VIP score analysis, the major ginsenoside metabolites in RG included MG-Rb1, MG-Rb2, vina-ginsenoside R8, NG-R1, NG-R4, G-Rg1, G-Re, G-Ra1, G-Ra2, G-Rb1, and G-Rd. In contrast, the ginsenoside metabolites distinguishing BG from RG were gypenoside XVII, G-Rg5, G-Rs3, and G-Rk1, in that order. RG undergoes a single steaming and drying process, leading to the partial hydrolysis of major ginsenosides. Unlike BG, RG is steamed once and then dried, so some major ginsenosides are hydrolyzed. However, malonyl ginsenoside was present in trace amounts.

## 4. Discussion

BG is reported to contain significant amounts of 5-HMF and melanoidin components due to the repeated steaming process. These compounds have been extensively documented in numerous articles for their robust antioxidant properties and potential cancer-preventing effects [27,28,29]. In this study, we discussed the formation of browning compounds through the action of reducing sugars and reducing amino acids. We confirmed that maltose, glucose, and fructose were the key substances in major browning reactions involving L-arginine, L-histidine, and L-lysine. Phenolic compounds have been reported to increase during the repetitive steaming process, with maltol playing a crucial role as the primary antioxidant. This suggests that maltol can serve as a significant metabolic marker for differentiating BG from conventional processed ginseng [30].

A precise structural elucidation method for BG’s ginsenoside metabolites in the positive ion mode was provided in this study. It allows for the accurate confirmation of adduct ion patterns and the patterns of glycoside detachment in the saponin structures. The major differentiating ginsenoside between WG and BG was malonyl ginsenoside. Malonyl ginsenosides refer to acidic ginsenosides in which the carboxyl group of malonic acid is esterified with neutral ginsenosides. Malonyl ginsenosides were reported to constitute 35–60% of the total ginsenoside content in unprocessed Korean ginseng [31].

**Figure 9:**
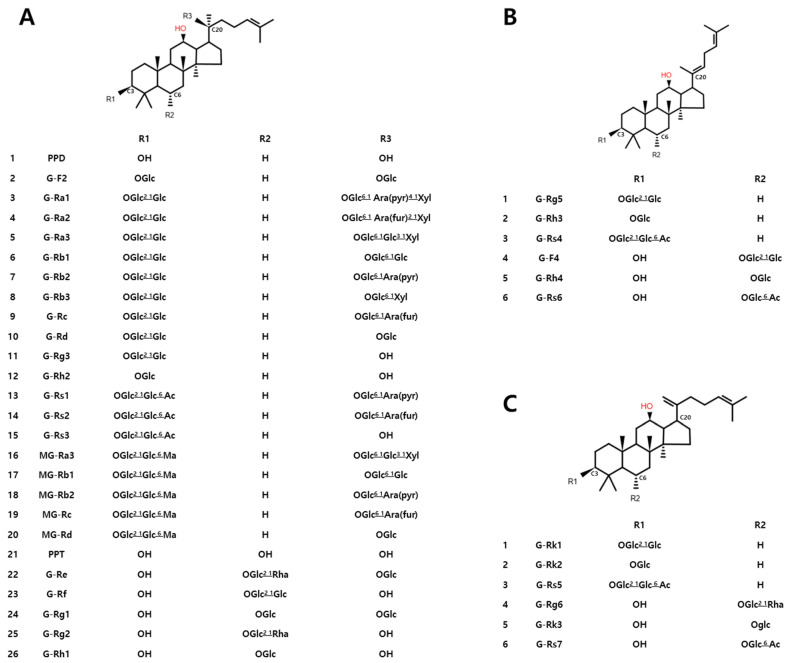
Major ginsenoside structures during the black ginseng manufacturing process; (**A**): Protopanaxadiol/Protopanaxatriol; (**B**): (E)-3β,6α,12β-trihydroxydammar-20(22),24-diene; (**C**): 3β,6α,12β-trihydroxydammar-20(21),24-diene; G: Ginsenoside; M: malonyl; Glc: β-D-Glucopyranosyl; Rha: α-L-Rhamnopyranosyl; Ara(fur): α-L-arabinofuranosyl; Ara(pyr): α-L-arabinopyranosyl; Xyl: β-D-xylopyranosyl; Ac: acetyl.

The primary difference between RG and BG lies in the concentration of minor ginsenosides, primarily attributed to the number of steaming cycles. The structural characteristics related to this are shown in Figure 9.

During the production of BG, protopanaxadiol (PPD)-type G-Rb1 and G-Rc undergo hydrolysis reactions, leading to the removal of the glycosyl moiety at C-20, resulting in their conversion to G-Rg3. Subsequently, G-Rg3 is further transformed into G-Rk1 and G-Rg5 through hydrolysis. In the case of protopanaxatriol (PPT)-type saponins, G-Re loses one glucose molecule at C-20, becoming G-Rg2, which further transforms into G-Rg4 and G-Rg6 through additional hydrolysis. This repetitive steaming process leads to a significant increase in the concentration of less polar ginsenosides. [32,33,34].

BG is rich in low-polarity ginsenosides compared to other processed ginseng types, making it a promising candidate for future functional food applications.

## 5. Conclusions

This study confirmed that during the processing of BG, the major factors responsible for the changes in its appearance are reducing sugars and reducing amino acids, which serve as key precursors for the formation of browning compounds. These elements can be utilized as significant metabolic markers to distinguish ginsenosides in ginseng species and processed ginseng products.

A method for the structural elucidation of ginsenosides using UPLC-QTOF-MS in the positive ion mode and the differentiation of ginsenoside metabolites was presented. This represents a novel approach with the potential for application in various research fields.

BG is abundant in less polar ginsenosides, setting it apart from other processed ginseng types. This characteristic enhances its potential value as a future functional food ingredient.

## Figures and Tables

**Table 1:** Free sugar content by black ginseng steaming process.

Free Sugar	Steaming Times		
	2022 MWG	2022 MRG	2022 MBG
Rhamnose	N.D	N.D	87.42 ± 6.96
Xylose	N.D	N.D	20.38 ± 2.78 ****
Fructose	2.71 ± 0.25	2.81 ± 0.24	32.31 ± 2.65 ****
Glucose	3.06 ± 0.27	3.18 ± 0.34	19.16 ± 0.86 ****
Sucrose	147.05 ± 1.65	74.32 ± 1.89 ****	10.28 ± 0.56 ****
Maltose	82.43 ± 4.01	76.75 ± 7.45	10.74 ± 1.02 ****

The data are expressed as the mean ± SD (n = 3); **** *p* < 0.0001, compared to 2022 WG.

**Table 2:** Free amino acid content by black ginseng steaming process (Unit: mg/g).

Free Amino Acid	2022-MWG	2022-MRG	2022-MBG
L-alanine	4.68 ± 0.19	5.19 ± 0.26	4.92 ± 0.26
L-Arginine	15.49 ± 0.37	16.71 ± 0.68 *	5.24 ± 0.12 ****
L-Aspartic acid	7.96 ± 0.51	7.81 ± 0.41	6.63 ± 0.30 *
L-Cystine	0.95 ± 0.04	0.94 ± 0.05	0.63 ± 0.02 ***
L-Glutamic acid	7.10 ± 0.05	4.68 ± 0.10 ****	4.76 ± 0.09 ****
Glycine	3.06 ± 0.15	3.05 ± 0.05	3.10 ± 0.25
L-Histidine	3.07 ± 0.04	3.05 ± 0.05	1.91 ± 0.02 ****
L-Isoleucine	4.84 ± 0.07	5.03 ± 0.13	4.78 ± 0.09
L-Leucine	2.33 ± 0.09	2.35 ± 0.11	2.23 ± 0.01
L-Lysine	4.23 ± 0.09	3.15 ± 0.08 ****	0.97 ± 0.01 ****
L-Methionine	0.68 ± 0.04	0.84 ± 0.06 *	0.86 ± 0.05 **
L-Phenylalanine	3.67 ± 0.04	3.82 ± 0.07 *	3.63 ± 0.03
L-Proline	2.97 ± 0.05	2.68 ± 0.03 ***	2.57 ± 0.05 ***
L-Serine	3.26 ± 0.13	3.35 ± 0.06	3.07 ± 0.06
L-Threonine	4.23 ± 0.09	4.25 ± 0.16	3.91 ± 0.08 *
L-Tyrosine	2.88 ± 0.09	2.99 ± 0.03	2.38 ± 0.09 ***
L-Valine	2.87 ± 0.04	2.83 ± 0.04	2.75 ± 0.04 *

The data are expressed as the mean ± SD (n = 3); * *p* < 0.05, ** *p* < 0.01, *** *p* < 0.001, **** *p* < 0.0001, compared to 2022 WG.

**Table 3:** Identification of ginsenoside compounds by UPLC-QTOF-MS analysis in positive ion mode.

Peak No.	t_R_ (min)	Identified Compounds	Molecular Formula	Theoretical Exact Mass (Da)	Precursor Ion/or Adduct Ions	Mean Measured Mass (Da)	Mass Error (ppm)
1	9.65	Ginsenoside Re4	C_47_H_80_O_18_	933.5417	933.5417[M+H^+^], 955.5236[M+Na^+^]	955.52463	1
2	9.68	Notoginsenoside R3 isomer	C_48_H_82_O_19_	962.5450	963.5523[M+H^+^], 985.5342[M+Na^+^]	985.53504	0.8
3	9.97	20-O-glucoginsenoside Rf	C_48_H_82_O_19_	962.5450	985.5342[M+Na^+^]	985.5308	−3.5
4	10.52	Notoginsenoside R1	C_48_H_80_O_18_	932.5339	955.5236[M+Na^+^]	955.5202	−3.7
5	11.699	Ginsenoside Rg1	C_42_H_72_O_14_	800.4922	823.4814[M+Na^+^]	823.47802	−4.1
6	11.792	Ginsenoside Re	C_48_H_82_O_18_	946.5501	947.5573[M+H^+^], 969.5393[M+Na^+^]	969.53561	−3.8
7	13.26	Malonyl-ginsenoside Rg1	C_45_H_74_O_17_	886.4926	887.4998[M+H^+^], 909.4818[M+Na^+^]	909.48202	0.2
8	14.292	Malonyl-ginsenoside Re	C_51_H_84_O_21_	1032.5500	1033.5577[M+H^+^], 1055.5397[M+Na^+^]	1055.54048	0.7
9	14.62	Vinaginsenoside R8	C_48_H_82_O_19_	962.5445	963.55231[M+H^+^], 985.53425[M+Na^+^]	985.5356	1.4
10	16.85	Vinaginsenoside R4	C_48_H_82_O_19_	962.5445	963.55231[M+H^+^], 985.53425[M+Na^+^]	985.53597	1.7
11	17.6	Vinaginsenoside R1	C_44_H_74_O_15_	842.5022	843.51005[M+H^+^], 865.49199[M+Na^+^]	865.49287	1
12	18.349	Pseudoginsenoside F11	C_42_H_72_O_14_	800.4922	801.4994[M+H^+^], 823.4814[M+Na^+^]	823.47777	−4.4
13	18.384	Ginsenoside Rf	C_42_H_72_O_14_	800.4922	801.4994[M+H^+^], 823.4775[M+Na^+^]	823.47753	−4.7
14	18.484	Pseudoginsenoside RT5	C_36_H_62_O_10_	654.4338	655.4415[M+H^+^], 677.4235[M+Na^+^]	677.42414	0.9
15	19.628	Notoginsenoside R2	C_41_H_70_O_13_	770.4816	771.48892[M+H], 793.47086[M+Na]	793.4676	−4.1
16	20.23	Notoginsenoside R4(s)	C_59_H_100_O_27_	1240.6452	1241.6524[M+H^+^], 1263.6344[M+Na^+^]	1263.62963	−3.8
17	20.9	Ginsenoside Rg2(s)	C_42_H_72_O_13_	770.4816	785.5045[M+H^+^], 807.4865[M+Na^+^]	807.48683	1.7
18	20.93	Ginsenoside Rh1(s)	C_36_H_62_O_9_	784.4973	785.5045[M+H^+^], 807.4865[M+Na^+^]	807.48683	0.4
19	20.965	Ginsenoside Rg2(r)	C_42_H_72_O_13_	638.4394	661.4286[M+Na^+^]	661.42859	0
20	21.34	Ginsenoside Rh1(r)	C_36_H_62_O_9_	784.4973	785.5045[M+H^+^], 807.4865[M+Na^+^]	807.4868	0.4
21	21.67	Ginsenoside F3	C_41_H_70_O_13_	770.4816	771.4889[M+H^+^]	771.48649	−3.1
22	21.83	Ginsenoside F1	C_36_H_62_O_9_	638.4394	639.4466[M+H^+^], 661.4286[M+Na^+^]	661.4286	1.9
23	21.87	Ginsenoside Ra2	C_58_H_98_O_26_	638.4394	1211.6419[M+H^+^], 1233.6238[M+Na^+^]	1233.62517	1.8
24	21.98	Ginsenoside Ra3	C_59_H_100_O_27_	1210.6346	1241.6524[M+H^+^], 1263.6344[M+Na]^+^	1263.63442	1.1
25	22.24	Ginsenoside F5	C_41_H_70_O_13_	1240.6452	771.48892[M+H], 793.47086[M+Na]	793.47221	1.7
26	22.32	Notoginsenoside R4(r)	C_59_H_100_O_27_	1240.6452	1241.6524[M+H^+^], 1263.6344[M+Na^+^]	1263.63638	1.6
27	22.396	Ginsenoside Rb1	C_54_H_92_O_23_	1108.6029	1109.6102[M+H^+^], 1131.5921[M+Na^+^]	1131.5928	0.6
28	22.95	Malonyl Ginsenoside Ra3	C_62_H_102_O_30_	1326.6450	1327.6528[M+H^+^], 1349.6348[M+Na^+^]	1349.63545	0.5
29	23.13	Malonyl Ginsenoside Rb1	C_57_H_94_O_26_	1194.6033	1195.6106[M+H^+^], 1217.5925[M+Na^+^]	1217.59302	0.4
30	23.2	Ginsenoside Ro	C_48_H_76_O_19_	1078.5924	1079.5996[M+H^+^], 1101.5816[M+Na^+^]	1101.5813	−0.3
31	23.3	Ginsenoside Rc	C_53_H_90_O_22_	956.4981	957.5053[M+H^+^], 975.5397[M+H^+^+NH_4_^+^], 979.4873[M+Na^+^]	979.48822	0.9
32	23.468	Ginsenoside Ra1	C_58_H_98_O_26_	1210.6346	1211.6419[M+H^+^], 1233.6238[M+Na^+^]	1233.62397	0.1
33	23.985	Malonyl Ginsenoside Ra2/ra1	C_61_H_100_O_29_	1296.6345	1297.6423[M+H^+^], 1319.6242[M+Na^+^]	1319.62522	0.7
34	24.122	Malonyl Ginsenoside Rc	C_56_H_92_O_25_	1164.5928	1165.6000[M+H^+^], 1188.5898[M+H+Na^+^]	1188.58585	−3.3
35	24.345	Ginsenoside Rb2	C_53_H_90_O_22_	1078.5924	1079.5996[M+H^+^], 1101.5816[M+Na^+^]	1101.58222	0.6
36	24.515	Malonyl ginsenoside Rb1 isomer	C_57_H_94_O_26_	1194.6028	1195.6106[M+H^+^], 1217.5925[M+Na^+^]	1217.59381	1
37	24.75	Pseudo ginsenoside RT1	C_47_H_74_O_18_	926.4870	927.49479[M+H^+^], 944.52134[M+NH_4_^+^]	944.52255	1.3
38	24.752	Ginsenoside Rb3	C_53_H_90_O_22_	1078.5924	1079.5996[M+H^+^], 1101.5816[M+Na^+^]	1101.58357	1.8
39	24.968	Malonyl Ginsenoside Rb3	C_56_H_92_O_25_	1164.5922	1165.6000[M+H^+^], 1187.5819[M+Na^+^]	1187.582	0
40	25.072	Malonyl Ginsenoside Rb2	C_56_H_92_O_25_	1164.5928	1165.6000[M+H^+^], 1187.5819[M+Na^+^]	1187.58208	0.1
41	25.433	Malonyl Ginsenoside Rb3 isomer	C_56_H_92_O_25_	1164.5922	1165.6000[M+H^+^], 1187.5819[M+Na^+^]	1187.582	0
42	26.372	Ginsenoside Rd	C_48_H_82_O_18_	946.5501	947.5573[M+H^+^], 969.5393[M+Na^+^]	969.54125	2
43	26.616	Ginsenoside Rs1	C_55_H_92_O_23_	1120.6024	1121.6102[M+H^+^], 1143.5921[M+Na^+^]	1143.59365	1.3
44	27.07	Malonyl Ginsenoside Rd isomer	C_51_H_84_O_21_	1032.5505	1033.5577[M+H^+^], 1055.5397[M+Na^+^]	1055.5406	0.8
45	27.47	Malonyl Ginsenoside Rd	C_51_H_84_O_21_	1032.5500	1033.5577[M+H^+^], 1055.5397[M+Na^+^]	1055.53985	0.1
46	27.607	Ginsenoside Rs2	C_55_H_92_O_23_	1120.6024	1121.6102[M+H^+^]	1121.60602	−3.7
47	28.359	Gypenoside XVII	C_48_H_82_O_18_	946.5501	969.5393[M+Na^+^]	969.5398	0.5
48	29.48	Ginsenoside Ra7	C_57_H_94_O_23_	1146.6180	1147.6258[M+H^+^]	1169.60771	−0.1
49	29.712	Notoginsenoside Fe	C_47_H_80_O_17_	916.5390	917.5468[M+H^+^], 939.5287[M+Na^+^]	939.51939	−10
50	30.486	Ginsenoside Rg6	C_42_H_70_O_12_	766.4867	767.4940[M+H^+^], 789.4759[M+Na^+^]	789.47806	2.7
51	30.498	Ginsenoside Ra8	C_57_H_94_O_23_	1146.6180	1147.6258[M+H^+^], 1169.6078[M+Na^+^]	1169.60795	0.1
52	30.695	Vinaginsenoside R16	C_47_H_80_O_17_	916.5390	917.5468[M+H^+^], 939.5287[M+Na^+^]	939.5298	1.1
53	31.17	Ginsenoside Rd2	C_47_H_80_O_17_	916.5390	917.54683[M+H^+^], 939.52877[M+Na]	939.53091	2.3
54	31.18	Ginsenoside Rg4	C_42_H_70_O_12_	766.4867	767.4940[M+H^+^], 789.4759[M+Na^+^]	789.47808	2.7
55	31.23	Gypenoside L	C_42_H_72_O_14_	800.4922	801.4994[M+H^+^], 823.4814[M+Na^+^]	823.47816	−4
56	31.51	Ginsenoside Rk3	C_36_H_60_O_8_	620.4288	621.4361[M+H^+^], 643.4180[M+Na^+^]	643.41944	2.2
57	32.488	Ginsenoside Rh4	C_36_H_60_O_8_	620.4288	621.4361[M+H^+^], 643.4180[M+Na^+^]	643.42013	3.2
58	32.83	Vinaginsenoside R3	C_48_H_82_O_17_	930.5547	931.56248[M+H^+^], 953.54442[M+Na^+^]	953.54494	0.5
59	33.7	Ginsenoside F2	C_42_H_72_O_13_	784.4973	785.5045[M+H^+^], 807.4865[M+Na^+^]	807.4893	3.5
60	33.98	Ginsenoside Z-R1	C_42_H_66_O_14_	794.4447	812.47908[M+NH_4_^+^], 817.43448[M+Na^+^]	812.48052	1.8
61	34.865	3-Acetyl-ginsenoside F1	C_38_H_64_O_10_	680.4494	681.45722[M+H^+^], 703.43917[M+Na^+^]	703.44024	1.5
62	36.068	Ginsenoside Rg3(s)	C_42_H_72_O_13_	784.4973	785.5045[M+H^+^], 807.4865[M+Na^+^]	807.48781	1.6
63	36.749	Ginsenoside Rg3(r)	C_42_H_72_O_13_	784.4973	785.5045[M+H^+^], 807.4865[M+Na^+^]	807.48771	1.5
64	37.115	Protopanaxatriol	C_30_H_52_O_4_	476.3866	477.3938[M+H^+^], 499.3757[M+Na^+^]	499.37701	2.5
65	39.19	Ginsenoside MC	C_41_H_70_O_12_	754.4867	755.4940[M+H^+^], 777.4759[M+Na^+^]	777.47753	2
66	40.061	Ginsenoside CY	C_41_H_70_O_12_	754.4867	755.4940[M+H^+^], 777.4759[M+Na^+^]	777.47797	2.6
67	40.79	Ginsenoside Rs3	C_44_H_74_O_14_	826.5073	827.51513[M+H^+^], 849.49708[M+Na^+^]	849.49804	1.1
68	41.56	Ginsenoside Rs4	C_44_H_72_O_13_	808.4967	809.50457[M+H^+^], 831.48651[M+Na^+^]	809.50558	1.2
69	43.59	Ginsenoside Rk1	C_42_H_70_O_12_	766.4867	767.4940[M+H^+^], 789.4759[M+Na^+^]	789.47753	2
70	44.786	Ginsenoside Rg5	C_42_H_70_O_12_	766.4867	767.4940[M+H^+^], 789.4759[M+Na^+^]	789.47768	2.2
71	45.863	Ginsenoside CK	C_36_H_62_O_8_	622.4445	623.4517[M+H^+^], 645.4336[M+Na^+^]	645.43508	2.2
72	47.211	Ginsenoside Rh2(s)	C_36_H_62_O_8_	622.4445	623.4517[M+H^+^], 645.4336[M+Na^+^]	645.4345	1.3
73	47.798	Ginsenoside Rh2(r)	C_36_H_62_O_8_	622.4445	623.4517[M+H^+^], 645.4336[M+Na^+^]	645.43508	2.2
74	48.11	Pseudo ginsenoside Rh2	C_36_H_62_O_8_	622.4439	623.45175[M+H]^+^, 645.43369[M+Na^+^]	645.43447	1.2
75	49.34	Ginsenoside Rs5	C_44_H_72_O_13_	808.4967	809.50457[M+H^+^], 831.48651[M+Na^+^]	831.48782	1.6
76	52.171	Ginsenoside Rk2	C_36_H_60_O_7_	604.4339	627.4231[M+Na^+^]	627.42291	−0.3
77	52.62	Ginsenoside Rh3	C_36_H_60_O_7_	604.4339	627.4231[M+Na^+^]	627.42329	0.3
78	56.123	Protopanaxadiol	C_30_H_52_O_3_	460.3916	461.3989[M+H^+^], 483.3808[M+Na^+^]	483.3809	0.1
79	60.9	Ginsenoside Rs6/Rs7	C_38_H_62_O_9_	662.4388	663.4466[M+H^+^], 685.4286[M+Na^+^]	685.43639	11.4

## Data Availability

The data presented in this study are available upon request from the corresponding author. Data is not publicly available due to privacy.

## References

[1] Nam K.-Y. (2005). The comparative understanding between red ginseng and white ginseng processed ginsengs (panax ginseng C.A. Meyer). J. Ginseng Res..

[2] Jo E.-J., Kang S.-J., Kim A.-J. (2009). Effects of Steam-And Dry-Processing Temperatures on the Benzo(a)pyrene Content of Black and Red Ginseng. Korean J. Food Nutr..

[3] Ko S.-R., Choi K.-J., Han K.-W. (1996). Comparison of proximate composition, mineral nutrient, amino acid and free sugar contents of several panax species. Korean J. Ginseng Sci..

[4] Hong H.D., Kim Y.C., Rho J.H., Kim K.T., Lee Y.C. (2007). Changes on physiological properties of Panax ginseng C. A. Meyer during repeated steaming process. J. Ginseng Res..

[5] Nam K.Y., Lee N.R., Moon B.D., Song G.Y., Shin H.S., Choi J.E. (2012). Changes of Ginsenoside and Color from Black Ginseng Prepared by Steaming-Drying Cycles. Korean J. Med. Sci..

[6] Chang J.-K., Park C.-K., Shim K.-H. (2003). Chang in chemical components of red ginseng processed from the fresh ginseng stored at low temperature. Korean J. Food Preserv..

[7] Jin Y., Kim Y.J., Jeon J.N., Wang C., Min J.W., Jung S.Y., Yang D.C. (2012). Changes of ginsenosides and physiochemical properties in ginseng by new 9 repetitive steaming and drying process. Korean J. Plant Res..

[8] Kim E.J., Jung I.H., Van Le T.K., Jeong J.J., Kim N.J., Kim D.H. (2013). Ginsenosides Rg5 and Rh3 protect scopolamine-induced memory deficits in mice. J. Ethnopharmacol..

[9] Francis G., Kerem Z., Makkar H.P., Becker K. (2002). The biological action of saponins in animal systems: A review. Br. J. Nutr..

[10] Lee H., Park D., Yoon M. (2013). Korean red ginseng (Panax ginseng) prevents obesity by inhibiting angiogenesis in high fat diet-induced obese C57BL/6J mice. Food Chem. Toxicol..

[11] Kim H.J., Lee J.Y., You B.R., Kim H.R., Choi J.E., Nam K.Y., Moon B.D., Kim M.R. (2011). Antioxidant activities of ethanol extracts from black ginseng prepared by steamingdrying cycles. J. Korean Soc. Food Sci. Nutr..

[12] Metwaly A.M., Lianlian Z., Luqi H., Deqiang D. (2019). Black Ginseng and Its Saponins: Preparation, Phytochemistry and Pharmacological Effects. Molecules.

[13] Nam K.-Y., Kim Y.-S., Shon M.-Y., Park J.-D. (2015). Recent advances in studies on chemical constituents and biological activities korean black ginseng (panax ginseng C.A. Meyer). Korean J. Pharmacogn..

[14] (2023). FOOD CODE, Chapter 8 General Test Methods, 9. Analysis Method for Hazardous Substances in Food, 9.5 Benzo[a]pyrene, Ministry of Food and Drug Safety, Korean. https://various.foodsafetykorea.go.kr/fsd/#/.

[15] Oh H.B., Lee J.W., Lee D.E., Na S.C., Jeong D.E., Hwang D.I., Kim Y.S., Park C.B. (2021). Characteristics of Black Ginseng (Panax ginseng C.A. Mayer) Production Using Ginseng Stored at Low Temperature after Harvest. Metabolite.

[16] Herbert A.S. (1980). Handbook of Biochemistry.

[17] Park H.W., In G., Kim J.H., Cho B.G., Han G.H., Chnag I.M. (2014). Metabolomic approach for discrimination of processed ginseng genus (Panax ginseng and Panax quinquefolius) using UPLC-QTOF MS. J. Ginseng Res..

[18] Maillard L.C. (1912). Formation of melanoidins in a methodical way. Compt. Rend..

[19] Kim H.J., Jo J.S., Nam S.H., Park S.H., Mheen K.C. (1983). Free sugar distribution in ginseng plant and change of it’s content in the root with dehydration. Korean J. Ginseng Sci..

[20] Jee H.K., Cho Y.J., Kim C.T., Jang Y.S., Kim C.J. (2006). Increase of Solubility of Ginseng Radix by Extrusion Cooking. Korean J. Food Sci. Technol..

[21] Shin J.Y., Choi E.H., Wee J.J. (2001). New methods for separation of crude ginseng saponins. Korean J. Food Sci. Technol..

[22] Do J.-H., Kim S.-D., Oh H.-I., Hong S.-K. (1982). Effect of Sugars, Amino acids and Inorganic Nitrogenous Compounds on the Acceleration of Browning in Ginseng. J. Korean Agric. Chem. Soc..

[23] Sung Y.J., Park C.J., Kim B.R., Shin S.J. (2013). Conversion of Fructose to 5-HMF (5-hydroxymethylfurfural) in DMSO (dimethylsulfoxide) solvent. J. Korea TAPPI.

[24] Nakagawa K., Maeda H., Yamaya Y., Tonosaki Y. (2020). Maillard Reaction Intermediates and Related Phytochemicals in Black Garlic Determined by EPR and HPLC Analysis. Molecules.

[25] Worley B., Powers R. (2016). PCA as a practical indicator of OPLS-DA model reliability. Curr. Metabolomics.

[26] Banerjee P., Ghosh S., Dutta M., Subramani E., Khalpada J., RoyChoudhury S., Chakravarty B., Chaudhury K. (2013). Identification of Key Contributory Factors Responsible For Vascular Dysfunction in Idiopathic Recurrent Spontaneous Miscarriage. PLoS ONE.

[27] Oh H.-L., Kim C.-R., Kim N.-Y., Jeon H.-L., Doh E.-S., Kim M.-R. (2013). Characteristics and antioxidant activities of rehmanniae radix powder. J. Korean Soc. Food Sci. Nutr..

[28] Shahidi F., Wanasundara P.K. (1992). Phenolic antioxidants. Crit. Rev. Food Sci. Nutr..

[29] Lim J.H., Wen T.C., Matsuda S., Tanaka J., Maeda N., Peng H., Aburaya J., Ishihara K., Sakanaka M. (1997). Protection of is chemic hippocampal neurons by ginsenoside Rb1, a main ingredient of ginseng root. Neurosci. Res..

[30] Han B.-H., Park M.-H., Han Y.-N., Suh D.-Y. (1992). Chemical and biochemical studies on non-saponin constituents of korean ginseng. Korean J. Ginseng Sci..

[31] Kim H.-J., Jo J.-S. (1984). Studies on the physicochemical properties of korean ginseng (panax ginseng, C.M. Meyer) root starch. Korean J. Ginseng Sci..

[32] Yoon D.H., Shin W.C., Lee Y.-S., Kim S.M., Baek M.-I., Lee D.Y. (2020). A Comparative Study on Processed Panax ginseng Products using HR-MAS NMR-Based Metabolomics. Molecules.

[33] Jang J.K., Shim K.H. (1994). Physicochemical Properties of Freeze Dried Ginseng from the Fresh Ginseng at Low Temperature. Korean J. Ginseng Sci..

[34] Kim M.H., Hong H.D., Kim Y.C., Rhee Y.K., Kim K.T., Rho J.H. (2010). Ginsenoside Changes in Red Ginseng Manufactured by Acid Impregnation Treatment. J. Ginseng Res..

